# Single‐Embryo Proteomics Reveals the Impact of Bovine Oviductal Spheroids on Individual Blastocyst Proteomes

**DOI:** 10.1002/mrd.70156

**Published:** 2026-11-15

**Authors:** Coline Mahé, Daniel Tomas, Valérie Labas, Joanna M. G. Souza‐Fabjan, Marie Saint‐Dizier

**Affiliations:** ^1^ INRAE, CNRS University of Tours Nouzilly France; ^2^ INRAE, CHU of Tours, PIXANIM Platform (Phenotyping & in/ex vivo Imaging From Animal to the Molecule) University of Tours Nouzilly France; ^3^ Faculdade de Veterinária Universidade Federal Fluminense—UFF Niterói Rio de Janeiro Brazil

**Keywords:** cattle, oviduct, proteomic, single embryo, spheroid

## Abstract

Our objective was to highlight the potential of single‐embryo proteomics for studying embryo‐maternal interactions. Single bovine blastocysts were analyzed by nanoLC‐MS/MS after co‐culture or not (controls) with isthmic epithelial spheroids (IES) for 5 days. A total of 3326 proteins were identified, among which 782 were differentially abundant following IES exposure. Notably, 617 DAPs (79%) were overabundant compared to controls, including 45 predicted to be secreted by IES and potentially internalized by embryos. This work provides a new methodology for studying embryo‐oviduct interactions while accounting for individual embryonic responses.

## 1 Introduction

In cattle, in vitro‐produced embryos are widely used for genetic selection, although their quality remains inferior to that of in vivo‐derived ones (Lonergan 2007). This discrepancy may stem from the absence of the maternal environment, particularly during the critical early stages of embryo development. The bovine embryo spends its first 4–5 days in the isthmus, where it undergoes its first mitotic divisions and major activation of its genome, at the 8‐cell stage, before entering the uterus (Banliat et al. 2022a; Graf et al. 2014; Hamilton 1946). Mimicking the oviduct environment in vitro may improve in vitro outcomes and offer valuable insights into embryo‐maternal dialogue. Compared to oviduct epithelial cell (OEC) monolayers, isthmic epithelial spheroids (IES) are apical‐out 3D models that retain key epithelial properties when cultured in synthetic oviduct fluid (SOF) medium (Pranomphon et al. 2024). Co‐culture of embryos with IES has been shown to improve blastocyst rates and change the transcriptome of expanded blastocysts compared to embryos cultured alone (Pranomphon et al. 2024). In addition, previous proteomic studies showed that bovine in vivo‐derived early embryos internalize proteins originating from the oviduct fluid, including oviductin (OVGP1) (Banliat et al. 2022b). However, there is scarce data on the proteomic changes induced by the oviductal epithelium in developing embryos. Furthermore, proteomic analyses of embryos have typically relied on pooled samples of at least four embryos per replicate (Banliat et al. 2022a, 2022b), precluding any evaluation of interindividual variability.

Therefore, this study aimed at characterizing the effect of an exposure to IES during the first 5 days of development on the blastocyst proteome using a single embryo proteomic approach.

## 2 Materials and Methods

All cultures were performed in a humidified atmosphere at 38.5°C with 5% CO_2_ in air.

The culture and selection of IES from early postovulatory oviducts were performed as previously described (Mahé et al. 2025). Briefly, for each replicate, the isthmus was isolated from 3 to 4 pairs of oviducts at the postovulatory stage collected at a local slaughterhouse. Isthmic segments were isolated from surrounding tissues and mucosal cells were expelled by pressure into 10 mL of culture medium (M199 + 2.5% HEPES + 0.5% gentamycin + 10% fetal calf serum [FCS]), vortexed for 1 min and left to sediment for 10 min. This vortex‐sedimentation step was repeated once, and cells were cultured at a 1:100 dilution. After 4 days of culture, 120 IES were selected based on their size (100–150 µm), presence of an internal cavity, and outward ciliary beating. One day later, i.e. twenty‐four hours before embryo culture, 100 IES were selected and washed in SOF with 5% FCS (SOF‐FCS), then transferred by groups of 25 into 20‐µL SOF‐FCS droplets under oil in a four‐well plate.

In vitro embryo production was performed as previously described with slight modifications (Pranomphon et al. 2024; Pranomphon et al. 2024). Following follicular fluid aspiration, cumulus‐oocyte complexes (COCs) with three or more cumulus cell layers were washed in EuroFlush (IMV Technologies), and transferred by groups of 50 in 500 µL maturation medium (M199 with 100 µM cysteamine, 1 µM resveratrol, 1.6 nM EGF, and 1.5 µM bovine serum albumine [BSA]) for 22 h. For in vitro fertilization (IVF, **Day 0**), two straws from one Holstein bull and one Normande bull (Synetics) were thawed at 38.5°C for 3 min, deposited onto a BoviPure–BoviDilute density gradient (40/80%; Nidacon), and centrifuged for 20 min at 700 × *g*. During centrifugation, COCs were washed in IVF medium (Parrish et al. 1988) and transferred into 500 µL of IVF medium in a 4‐well plate. The sperm pellet was resuspended in 1 mL of IVF medium and centrifuged at 300 × *g* for 10 min. After sperm counting, 1 × 10^6^ sperm/mL were incubated with 50 COCs in 500 µL of IVF medium for 22 h.

On Day 1, presumptive zygotes were vortexed for 2 min to remove cumulus cells, then washed twice with EuroFlush and once with SOF‐FCS. Twenty‐five zygotes were transferred in 5 µL into 20‐µL droplets of SOF‐FCS either with 25 IES (+IES) or alone (control). On Day 6, IES were removed using less than 5 µL, and embryos were cultured until Day 8. Each biological replicate was defined as one pool of COCs for embryo production and one pool of IES.

Cleavage rates were evaluated on Day 2 and blastocyst rates on Day 8 (expressed as a percentage of the total number of cleaved embryos at Day 2). Assessment of total cell numbers and proportion of cells in the inner cell mass (ICM) of Day‐8 blastocysts was performed as previously described (Pailles et al. 2022). Briefly, subgroups of expanded blastocysts were fixed (4% paraformaldehyde + 0.5% Triton X‐100 + 1% BSA) and immunostained with a rabbit polyclonal anti‐SOX2 antibody (4.5 µg/mL; Abcam ab97959) and Hœchst (2 µg/mL). The blastocysts were mounted between a slide and a coverslip and imaged using a confocal microscope. For each blastocyst, three to four Z‐stacks images were acquired, enabling the visualization of the entire embryo. Images were analyzed in Fiji to count the total number of cells (Hœchst+) and those composing the ICM (SOX2+). Five biological replicates were assessed.

For proteomic analysis, three Day‐8 expanded blastocysts of similar diameters per group were selected from the same IVF replicate according to the following morphological criteria: IETS grade 1, that is, a fully expanded blastocoel, a thinned zona pellucida, and a compact ICM with a cohesive trophectoderm (Figure 1B). Blastocysts were imaged using a microscope, rapidly washed twice in SOF without FCS, then three times in prewarmed (37°C) filtered PBS. Samples were then prepared using activated Evotips (Evosep) following the manufacturer's instructions. Briefly, 1.1 µL of 2X lysis buffer (iST kit, PreOmics) was deposited onto the wall of the Evotip, followed by 1 µL of PBS containing a single blastocyst and 2.1 µL of digestion buffer (iST kit, PreOmics). The Evotip box was then incubated at 37°C for 4 h to allow protein digestion. After Evotip washing and peptide elution, peptides were analyzed by nano‐liquid chromatography ion mobility‐tandem mass spectrometry (nanoLC‐IMS‐MS/MS) using an Orbitrap Ascend™ Tribrid™ high‐resolution mass spectrometer with a high‐field asymmetric waveform ion mobility spectrometry (FAIMS) coupled to a Vanquish Neo ultra‐high liquid chromatography system (Thermo Fisher). The mass spectrometer was operated in positive ion mode using data‐dependent acquisition. Raw data were analyzed in Proteome Discoverer™ 3.1.1.93 (Thermo Fisher Scientific) using the Chimerys 2.0 algorithm. Peptides and proteins were identified using a False Discovery Rate < 1%, a *Bos Taurus* database (UniprotBK v2025‐06‐18), and high confidence scoring (*q*‐value < 0.01). Proteins were identified with at least two different peptides (in terms of sequence or m/z).

**Figure 1:**
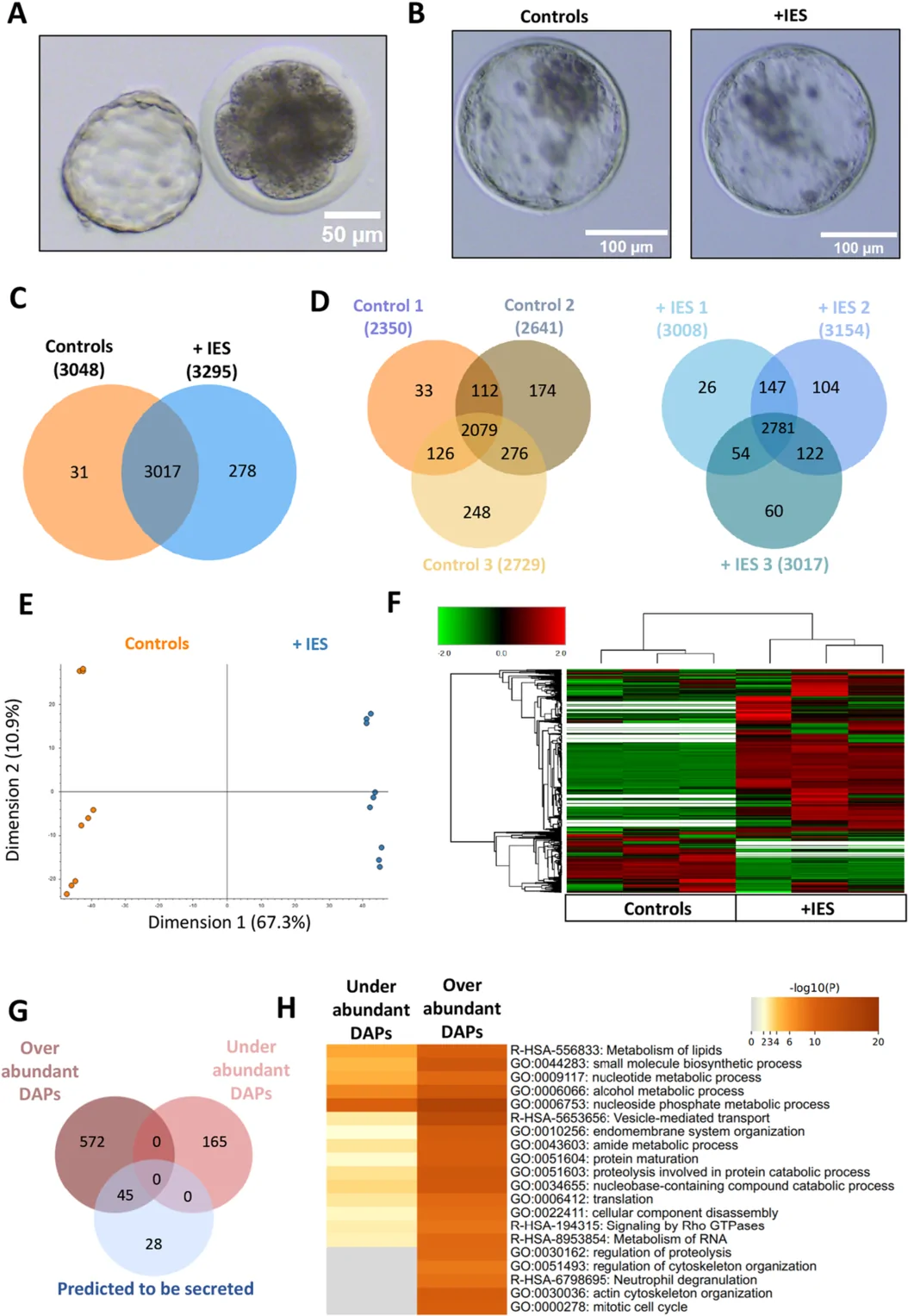
Proteomic changes in individual blastocysts after co‐culture with isthmic epithelial spheroids (IES). (A) Picture of an IES next to a 6‐cell embryo on Day 2 of culture; (B) Representative pictures of Day‐8 expanded blastocysts analyzed by nanoLC‐MS/MS. Embryos were exposed to IES for the first 5 days of culture (+IES) or cultured alone (Controls). (C) Venn diagram showing the overlap of all proteins identified between the control and +IES embryos. The number of proteins identified in each group is indicated in parentheses. (D) Venn diagrams of all proteins identified among individual embryos in the control and +IES groups. The number of proteins identified in each embryo is indicated in parentheses. (E) Principal component analysis of the 2946 proteins quantified in blastocysts. Three blastocysts per group from the same biological replicate were analyzed. One dot represents one technical replicate of one embryo. (F) Heatmap and unsupervised hierarchical clustering of differentially abundant proteins (DAPs) between the +IES and control embryos. Each line corresponds to one protein and each column to the normalized quantitative values of one embryo. Red indicates higher abundance while green indicates lower abundance compared with other conditions. (G) Venn diagram showing the overlap of DAPs that were either overabundant or underabundant in the +IES compared to control embryos, and the proteins predicted as secreted from IES. (H) Heatmap of the functional Metascape enrichment analysis presenting the list of Gene Ontology Biological Processes and Reactome pathways for overabundant and underabundant DAPs in +IES compared to control embryos.

Protein quantification was performed using a label‐free extracted ion chromatogram (XIC) approach based on pairwise ratios, with summed protein abundances normalized to the total peptide amount and modified peptides excluded from the analysis. Proteins were considered as quantifiable if they were identified with at least two different peptides with abundance values, including at least one unique peptide. Data were log‐transformed and statistically analyzed using Proteome Discoverer™ 3.1.1.93. The principal component analysis (PCA) and unsupervised hierarchical clustering were performed using quantifiable proteins. Differentially abundant proteins (DAPs) were identified using a background‐based *t*‐test (*p* ≤ 0.05, fold‐change ratio ≥ 2). This method uses the background population of ratios for all peptides and proteins to determine whether any single peptide or protein is significantly changing relative to that background. For more robust results, the Benjamini‐Hochberg correction were applied.

Functional enrichment analysis of DAPs was performed using Metascape (Sherman et al. 2022), the *Homo Sapiens* database, and Gene Ontology Biological Process and Reactome pathways.

Proteins were considered as potentially originating from the IES when they were (i) predicted to be secreted either through the presence of a signal peptide in their sequence or via an unconventional secretion pathway using OutCyte (Zhao et al. 2019); and (ii) detected in embryos co‐cultured with IES but not in control embryos.

Venn diagrams were carried out using jvenn (Bardou et al. 2014).

Data normality was assessed using the Shapiro–Wilk test. Embryo development rates and cell numbers were compared between groups using unpaired t‐tests (cleavage rates, blastocyst rates, total cell number and ICM cell proportions) or Mann–Whitney (hatching rates, diameter) tests in GraphPad Prism (*p*‐value ≤ 0.05). All data are presented as means ± standard error of the mean (SEM).

## 3 Results

No significant difference in rates of cleavage, blastocysts, total number of cells per expanded blastocyst, and proportion forming the ICM, was observed between groups (Table 1).

**Table 1:** Developmental rates and morphological data of blastocysts after exposure to isthmic epithelial spheroids (IES).

Day after IVF	Parameter	Controls	+IES	*p*‐value
Day 2	Cleavage rate (%)	79.1 ± 4.9	73.2 ± 1.7	0.3
Day 7	Blastocyst rate (%)	19.0 ± 3.7	18.2 ± 2.8	0.9
Day 8	Blastocyst rate (%)	25.1 ± 3.4	21.2 ± 4.4	0.5
	Hatching rate (%)	2.9 ± 2.9	16.2 ± 12.9	0.7
	Total cell number per expanded blastocyst	78.8 ± 8.3	97.0 ± 6.0	0.1
	Cells in the ICM (%)	28.9 ± 5.1	36.4 ± 2.0	0.2

*Note:* Data are represented as mean ± SEM of five biological replicates. Each biological replicate was defined as one pool of COCs for embryo production and one pool of IES. Blastocyst rates are expressed as a % of cleaved embryos.

Abbreviations: ICM, inner cell mass; IES, isthmic epithelial spheroids; IVF, in vitro fertilization.

The diameter of expanded blastocysts selected for nanoLC‐MS/MS analysis did not differ between groups: 176.4 ± 1.8 µm versus 190.9 ± 9.9 µm for controls and embryos exposed to IES, respectively (*n* = 3 per group from the same IVF; Figure 1B). Detailed proteomic data are provided in Supporting Information: Table S1. Overall, 3326 were identified in blastocysts, among which 278 exclusively after exposure to IES and 31 exclusively in controls (Figure 1C). Overall, 63% (2079/3326) and 84% (2781/3326) of identified proteins were shared among individual embryos in controls and after IES exposure, respectively (Figure 1D).

A total of 2946 proteins were quantified. The PCA of all proteins showed a close proximity of technical replicates within each individual embryo (Figure 1E). The mean coefficient of variation in protein abundance among technical replicates was 7.3 ± 0.3%. The PCA showed also a clear separation of samples according to the presence of IES (Figure 1E). The mean coefficients of variation in protein abundance among individual embryos were 11% and 12.4% for control and +IES groups, respectively. A total of 782 DAPs were evidenced between the +IES and control groups, of which 617 (79%) more abundant and 165 (21%) less abundant in the +IES group, as shown in the heatmap of DAPs (Figure 1F). Among the overabundant DAPs, 45 proteins, including oviductin (OVGP1), were predicted as secreted by IES and considered as candidate IES‐derived proteins (Figure 1G).

Functional analysis of the DAPs revealed significant enrichment in metabolic processes (metabolism of lipids, of nucleotides, amides…) as well as protein maturation and synthesis (metabolism of RNA, translation). Furthermore, processes such as the mitotic cell cycle, actin cytoskeleton organization, regulation of proteolysis and neutrophil degranulation were specifically associated with the proteins overabundant in the +IES group (Figure 1H).

## 4 Discussion

Using a single‐blastocyst approach, 3326 proteins were identified, representing the most comprehensive proteomic dataset in bovine embryos following co‐culture with IES. The high number of proteins identified and the low coefficient of variation among technical replicates demonstrate the high analytical reproducibility and robustness of the method reported here. Previous studies on bovine embryos using a similar nanoLC‐MS/MS approach identified between 1616 and 3028 proteins from pools of four (Banliat et al. 2022a, 2022b) to 25 (Banliat et al. 2020) embryos. Although differences in sample preparation, instrumentation, and data processing preclude a direct comparison of analytical sensitivity across studies, the comparable depth of proteome coverage from single embryos highlights the sensitivity and potential of the present workflow.

The main result of this study is that exposure to IES for the first 5 days of culture induced significant proteomic changes: 27% (782) of the quantified proteins exhibited altered abundance. These proteomic changes may primarily stem from the modulation of the embryonic genomic activation at the 8‐cell stage, leading to alterations in gene expression and protein translation. This is consistent with previous findings demonstrating that a 4‐day co‐culture with IES altered the expression of 559 genes in bovine blastocysts (Pranomphon et al. 2024). This also aligns with the functional analysis of DAPs, showing significant enrichment in RNA metabolism, protein translation, and maturation. Another possible mechanism underlying the proteomic changes in embryos is the direct internalization of IES‐derived proteins during culture, as previously demonstrated in bovine in vitro produced embryos co‐cultured with oviduct fluid (Banliat et al. 2020). This mechanism might play a significant role in embryo proteomic changes, as the majority of DAPs were overabundant after IES exposure. Among these were proteins known to be highly abundant in oviduct fluid, such as OVGP1 and CD109, which were part of the 45 candidate IES‐derived proteins. Once internalized by embryos, these proteins may contribute to support embryo development and quality, as previously reported for OVGP1 in cattle (Algarra et al. 2018; Choudhary et al. 2017). However, in our experimental design, the changes induced by soluble factors secreted from IES could not be distinguished from other sources of alterations, including oxygen consumption and changes to the culture medium caused by IES during co‐culture. Future experiments combining proteomic analyses of IES, IES‐conditioned medium, embryo–IES co‐culture medium, and individual embryos would provide a powerful approach to trace IES‐derived proteins and better distinguish their contribution to the embryonic proteome from other effects of the co‐culture environment.

In conclusion, this work provides a valuable resource for investigating how the oviduct shapes the embryonic proteome while accounting for individual embryonic variability. Future studies will build on these findings by analyzing larger populations of embryos at different developmental stages.

## Author Contributions

**Coline Mahé:** conceptualization, investigation, writing – original draft, writing – review and editing, methodology, validation, visualization, data curation. **Daniel Tomas:** methodology, formal analysis, software, resources, writing – review and editing, validation, data curation. **Valérie Labas:** funding acquisition, writing – review and editing, methodology, validation, software, resources, formal analysis, data curation; project administration. **Joanna M. G. Souza‐Fabjan:** writing – review and editing, validation. **Marie Saint‐Dizier:** conceptualization, investigation, funding acquisition, writing – original draft, writing – review and editing, methodology, validation, project administration, supervision, data curation.

## Conflicts of Interest

The authors declare no conflicts of interest.

## Supporting information


Supporting File

## Data Availability

The mass spectrometry proteomics data have been deposited to the ProteomeXchange Consortium via the PRIDE (Perez‐Riverol et al. 2025) partner repository with the dataset identifier PXD081612 and DOI: 10.6019/PXD081612 (https://www.ebi.ac.uk/pride/).
